# Fundoscopy in the smartphone age: current ophthalmoscopy methods in neurology

**DOI:** 10.1055/s-0043-1763489

**Published:** 2023-05-31

**Authors:** Richard Henrik Corr

**Affiliations:** 1 Universidade Federal do Rio de Janeiro, Hospital Federal dos Servidores do Estado, Rio de Janeiro RJ, Brazil.

**Keywords:** Ophthalmoscopy, Ophthalmoscopes, Smartphone, Oftalmoscopia, Oftalmoscópios, Smartphone

## Abstract

The observation of the human retina *in vivo* began in 1851 after the invention of the first ophthalmoscope by the German physicist Hermann von Helmholtz. In the following decades, direct and indirect ophthalmoscopy, with the use of ophthalmoscopes and condensing lenses, respectively, became part of the clinical examination, especially in ophthalmology and neurology. Today, over 170 years later, many ophthalmoscopes and condensing lenses exist on the market. Nevertheless, ophthalmoscopy is still not widely adopted as part of the physical exam of general practitioners, and the teaching of ophthalmoscopy in medical school remains challenging. Studies have shown that students prefer using newer ophthalmoscope models or condensing lenses during training, but most do not feel confident in performing ophthalmoscopy afterwards, regardless of the models used. Also, few students acquire ophthalmoscopes for their future practice, and clinical trials have not clearly demonstrated superiority of newer ophthalmoscope models over the conventional ones in diagnostic accuracy. The technological improvement of smartphone cameras in recent years has made it feasible to photograph the fundus of the eye using ophthalmoscopes or condensing lenses, reducing the need for retinographs and similar equipment. Smartphone assisted indirect fundoscopy is becoming increasingly popular. This approach allows adequate identification of the structures of the fundus, is cost-efficient, easy to implement, and permits easy recording and sharing of the images obtained, which is useful for case discussions and medical teaching. However, controlled clinical trials validating this method in the evaluation of optic nerve pathologies are needed.

## **Resumo**

A observação da retina humana *in vivo* começou em 1851, após a invenção do primeiro oftalmoscópio pelo físico alemão Hermann von Helmholtz. Nas décadas seguintes, a oftalmoscopia direta e indireta, com o uso de oftalmoscópios e lentes condensadoras, respectivamente, tornou-se parte do exame clínico, especialmente em oftalmologia e neurologia. Hoje, mais de 170 anos depois, diversos oftalmoscópios e lentes condensadoras estão disponíveis no mercado. No entanto, a oftalmoscopia ainda não é amplamente realizada no exame físico de médicos generalistas e o ensino da oftalmoscopia na faculdade de medicina ainda é desafiador. Estudos mostram que estudantes preferem usar modelos novos de oftalmoscópio ou lentes condensadoras durante seu treinamento, porém a maioria dos estudantes não se sente suficientemente confiante em realizar oftalmoscopia mais tarde, mesmo quando utilizam os modelos mais novos. Além disso, poucos alunos adquirem oftalmoscópios para sua prática futura, e ensaios clínicos não demonstraram uma clara superioridade dos novos modelos de oftalmoscópio em relação ao modelo convencional em acurácia diagnóstica. O aperfeiçoamento tecnológico das câmeras de smartphones nos últimos anos tornou possível fotografar o fundo do olho utilizando oftalmoscópios ou lentes condensadoras, reduzindo a necessidade de retinógrafos e equipamentos similares. A fundoscopia indireta realizada com smartphones vem se tornando cada vez mais popular. Esta abordagem permite a adequada identificação das estruturas do fundo do olho, é custo-efetiva, fácil de implementar e permite o fácil registro e compartilhamento das imagens obtidas, o que é útil para discussão de casos clínicos e para o ensino médico. Entretanto, ensaios clínicos controlados para a validação deste método na avaliação de patologias do nervo óptico são necessários.

## INTRODUCTION

### Origin of the ophthalmoscope

**Fig. 1:**
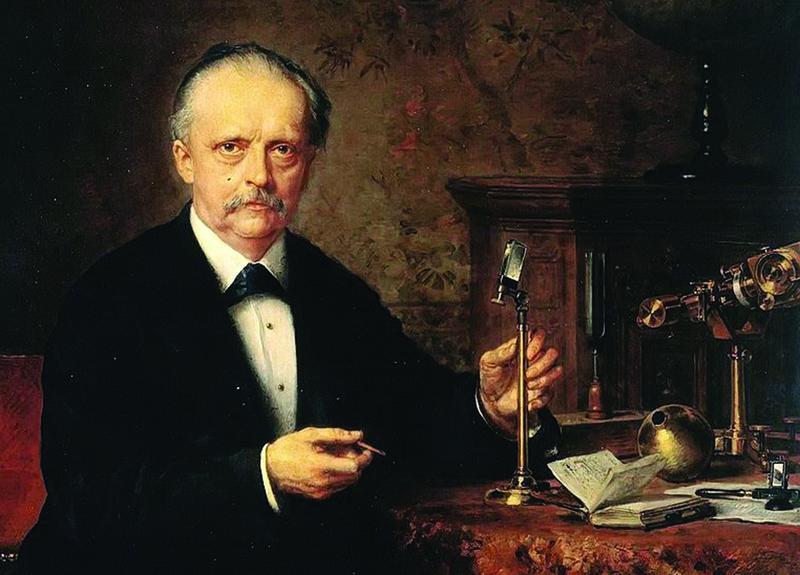
Hermann von Helmholtz in illustration by Ludwig Knaus, 1881. Helmholtz (1821–1894) was a physician, physiologist, and physicist. During his lifetime, he published many papers, theories, and textbooks. His most influential legacy in medicine is the creation of the first ophthalmoscope. Source: Knaus L., *Der Physiker Hermann von Helmholtz* (Portrait of the Physicist Hermann von Helmholtz) title QS:P1476,de:”Der Physiker Hermann von Helmholtz” [Internet]. 1881 [cited 2022 Apr 20]. **Available at:** https://commons.wikimedia.org/wiki/File:Hermann_von_Helmholtz_by_Ludwig_Knaus.jpg

The visualization of the internal structures of the eye has long been an idea surrounded by mystery and philosophy. However, the actual observation of the eye fundus *in vivo* as an auxiliary diagnostic method began in 1851, 28 years before the invention of the electric light bulb, when the German physicist, physician, and physiologist Hermann von Helmholtz ( Figure 1 ) presented a paper entitled “ *Augenspiegel* ” (“eye mirror”, in loose translation) to the Berlin Society of Physicists, where he described the functioning of what would later become known as the first ophthalmoscope. 1

Helmholtz noted that in order to be able to visualize the fundus of the eye *in vivo* with clarity, three problems had to be solved. First, refractive errors in both patient and examiner had to be corrected. Thus, a point on the patient's retina could, when illuminated, cast light rays that would focus on the observer's retina. Second, the patient's retina should be sufficiently illuminated. Third, the light source and the observer's eye should be aligned on the same optical axis; however, it was desirable that the light source did not interfere with the examination. 2

**Fig. 2:**
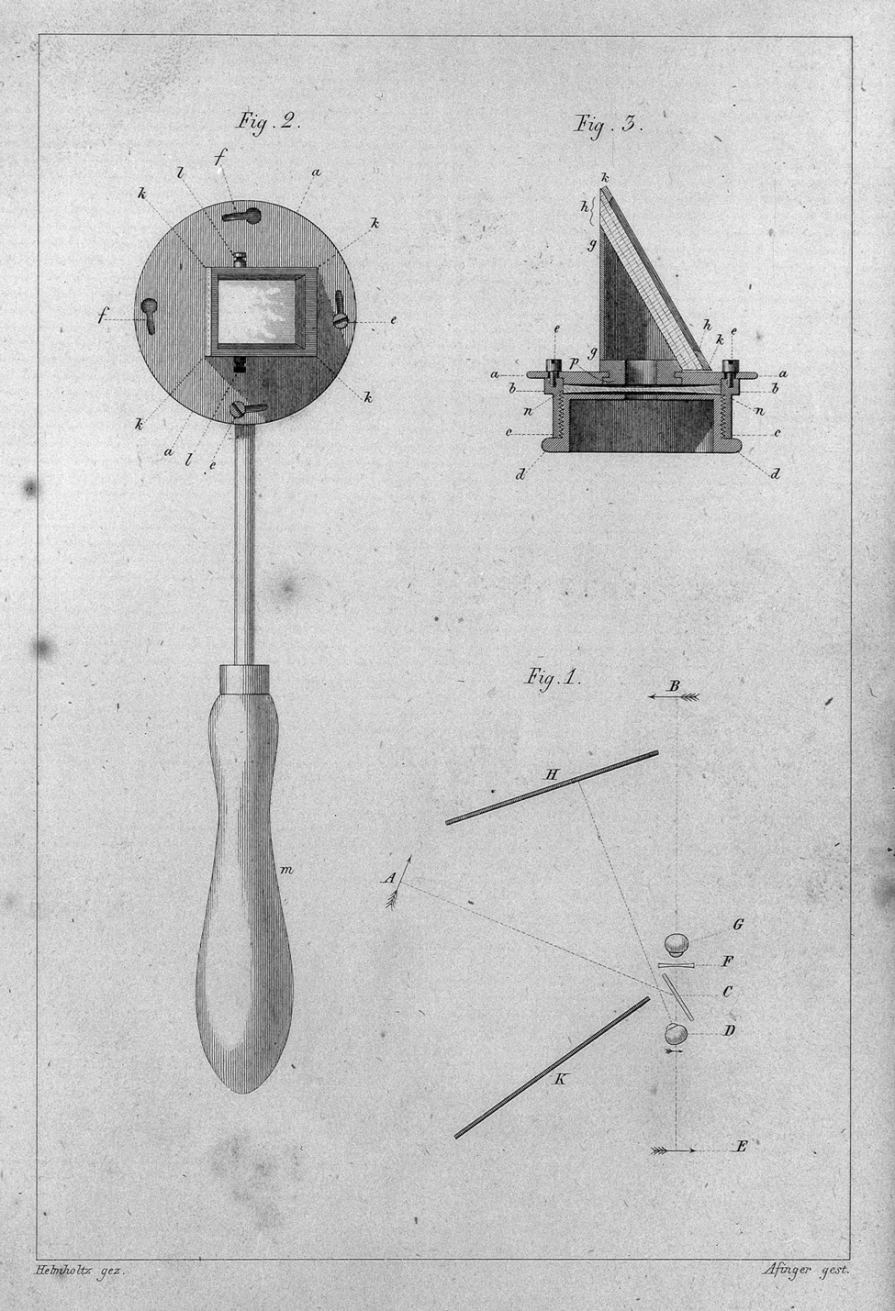
Depiction of the *Augenspiegel.* The first ophthalmoscope, initially named *Augenspiegel* , had a set of interchangeable lenses that slid into a slot in the eyepiece. The lenses should compensate for refractive errors of the eyes of both examiner and patient. The *Augenspiegel* 's eyepiece consisted additionally of three superimposed glass plates mounted obliquely to an opening through which the examiner would observe the patient's eye. The three layers of glass formed a reflective surface with some polarization capability, thereby reducing corneal reflection and allowing the adjacent light source, the patient's eye and the examiner's eye to be aligned on the same optical axis. Source: Helmholtz's ophthalmoscope [Internet]. Look and Learn History Picture Library. [cited 2022 Apr 20]. **Available at:** https://www.lookandlearn.com/history-images/YW010474M/Helmholtzs-ophthalmoscope

**Fig. 3:**
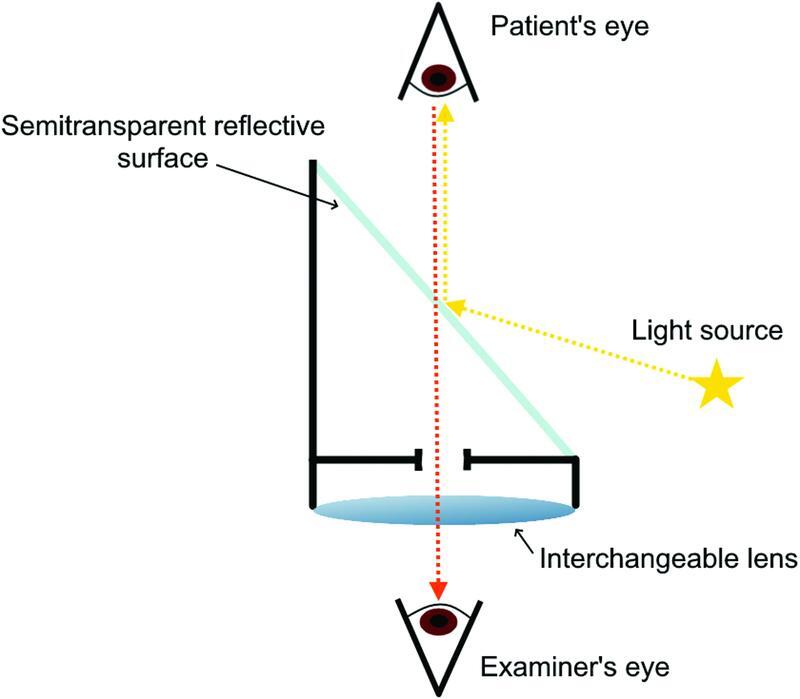
Optical principles of the *Augenspiegel.* Illustration of the optical principles used by *Augenspiegel* . The optical axis of the light source and the patient's retinal image must be aligned for the observer. **Source:** Elaborated by the author.

The first problem was overcome by using an interchangeable set of lenses placed inside of the ophthalmoscope ( Figures 2 and 3 ). A thinner or thicker lens would be used according to the refractive disturbances of the eyes of the patient and the examiner. The second problem, retinal illumination, in an age with no incandescent lamps, was solved initially with the use of a burning candle placed adjacently at an appropriate distance. The third problem, the optical alignment between light source, observer's eye, and examined eye, was a challenge that required creativity. Placing a candle or other light source directly between the observer and the patient was not adequate, since the intense shine of the flame would hinder the visualization of the fundus, while also not allowing the observer to get close to the examined eye. Helmholtz circumvented this problem by placing three superimposed layers of glass in front of the ophthalmoscope's window in an oblique arrangement relative to the light source, so that the glass would reflect the light rays coming from a nearby candle's flame toward the patient's eye, while also enabling the observation of the eye from the same optical axis through which the light was shining. 2 ( Figures 2 and 3 )

### Evolution of ophthalmoscopy

**Fig. 4:**
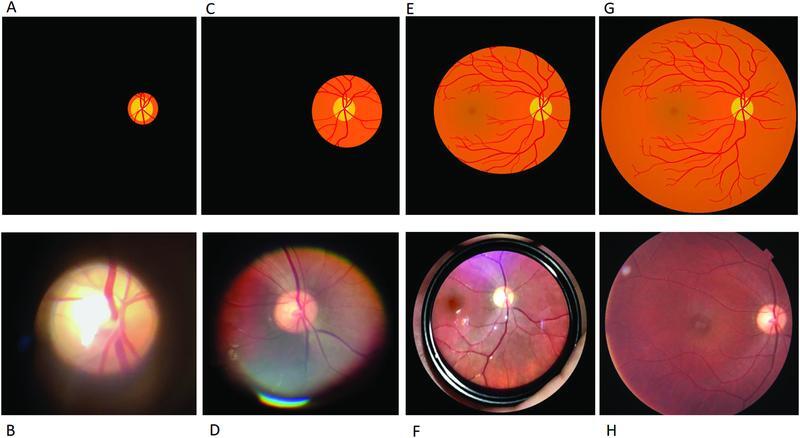
Comparison of digital images obtained through different ophthalmoscopy techniques, in schematic version (first line) and photographic examples (second line). **A** and **B** : conventional direct ophthalmoscope; **C** and **D** : PanOptic ophthalmoscope; **E** and **F** : indirect ophthalmoscopy using a 20 diopter condensing lens; **G** and **H** : retinographic. Image **F** was inverted vertically and horizontally to maintain similarity with the other images. **Sources:** **A** , **C** , **E** , **G** : Created by the author; **B** , **D** , **F** : Author's collection; **H** : Obtained by a DRI OCT Triton plus (TOPCON Corp., Tokyo, Japan) device. Images **B** , **D** , **F** , and **H** were obtained from the same patient after pupil dilation (with permission).

Helmholtz's invention soon gained popularity throughout Europe. New versions of the instrument were developed, gradually becoming more sophisticated. Eventually, the lighting source changed from candlelight to oil, gas combustion, and, finally, electric light. The reflecting surface gradually changed from superimposed glass plates into glass or metal mirrors, and later into prisms. The widely-used system of correcting lenses disposed in a rotating disk was introduced by Pickard and Curry in 1880. Today, hundreds of variations and improvements of the original model exist. 2 3 The modern direct ophthalmoscope provides a field of vision of up to 5 degrees with a magnification of 15x ( Figures 4 A and B) . The high magnification of this instrument makes it easy to identify and individually observe the structures at the eye fundus and to appreciate small changes therein, such as tiny new blood vessels, or dynamic events such as the venous pulsation at the optic disc. 3 4

While the ophthalmoscope was becoming popular, indirect ophthalmoscopy was refined as well. This method of fundoscopy also requires a light source that shines through the same optical axis as the observer and relies on the use of a condensing biconvex lens. Indirect ophthalmoscopy provides a true, horizontally and vertically inverted image of the retina. Its inclusion as a part of medical examination began one year after Helmholtz's publication, in 1852, under Theodor Ruete. 5 Almost one century later, in 1945, Charles Schepens had the idea of attaching the light source to his head, so that he could manipulate the lens with one hand and a scleral depressor with the other. This enabled the complete evaluation of the peripheral retina. 6 Indirect ophthalmoscopy has the advantage of providing a larger field of vision, according to the strength of the lens, but at the expense of lower image magnification when compared to a conventional ophthalmoscope. Condensing lenses of different diopters are available on the market, serving various purposes in ophthalmologic evaluation. Their use in conjunction with a binocular indirect ophthalmoscope enables a stereoscopic view of the fundus of the eye. The 20-diopter condensing lens (20D lens) is one of the most popular choices among neurologists, offering a 50° field of vision as well as 3x magnification. 7 ( Figures 4 E and F )

In the years following Helmholtz's creation, ophthalmoscopy enabled the *in vivo* detection of many pathologies for the first time. In 1853, Donders described retinitis pigmentosa. In 1860, von Graefe described papilledema, and in 1861, Jaeger first noted optic nerve atrophy. 2 4 Charcot himself was one of the first to promote ophthalmoscopy amongst neurologists, especially in doubtful diagnoses, stating: “Now, in these difficult times, the art of ophthalmoscopy has come to give us its decisive support” 4 8 . Today, ophthalmoscopy is considered fundamental to neurological examinations. This review aims to provide information regarding the impact of new technologies and methods in ophthalmoscopy, as well as their effect in the teaching and practicing of fundoscopy.

## METHODS

A literature search was performed in PubMed and Google Scholar using the keywords *ophthalmoscope* , *ophthalmoscopy* , *history* , *fundoscopy* , *smartphone* , *Arclight* , and *PanOptic* . Several studies and review articles were selected by the author's judgement according to historical accuracy, relevance of findings, date of publication and number of citations. The references of these articles were also scrutinized and, when considered relevant, incorporated into this review.

### Ophthalmoscopy in the Present Times

Notwithstanding the great value of fundoscopic evaluation by neurologists and nonspecialists, its practice in clinical examination and even the teaching of ophthalmoscopy in undergraduate medical education has shown a gradual decline in recent times. Mackay (2015) went so far as to claim that ophthalmoscopy is a “dying art.” 4 Indeed, performing ophthalmoscopy is challenging for both newly trained medical students and medical graduates, and even those who perform the exam on a regular basis can fail to detect abnormalities present in the fundus of the eye. 4 9 Thus, the emergence of new modalities of fundus evaluation, incorporating newer technologies, could rekindle the interest of undergraduates and medical doctors in ophthalmoscopy and, perhaps, facilitate the diagnosis of pathologies observable in the eye.

A 2009 article by Mottow-Lippa et al. evaluated the teaching of ophthalmoscopy in a medical school in California. The study, involving 84 students, showed that at the end of their academic training in ophthalmology, which was spread over a 3-year course, 99% of the students surveyed expressed a desire to receive further practical training. However, at graduation, only 13% of the students had purchased an ophthalmoscope, 9 suggesting a low rate of adoption of the fundus exam in the general medical examination. Possible reasons for this include the cost of the ophthalmoscope, especially for medical students, and the technical difficulty in performing direct ophthalmoscopy, since it requires a significant amount of training until the examiner feels sufficiently confident to perform it. 4 Another study by Kelly et al. (2013), involving over 100 undergraduate medical students, showed that students tended to prefer fundus evaluation through photographs over direct observation by ophthalmoscopy. This is also correlated with higher diagnostic accuracy among students in the group that assessed photographs, in comparison with the group that performed direct ophthalmoscopy. 10 This suggests that the restricted field of vision of the conventional ophthalmoscope can complicate the evaluation of the fundus of the eye by less experienced examiners.

In the last decades, two major new variations of conventional ophthalmoscopes were released in the market: The PanOptic (Welch Allyn, Inc., NY, USA) and the Arclight (University of St. Andrews, St. Andrews, Scotland). The PanOptic ophthalmoscope was first introduced to the medical community around the year 2004. 11 This model is larger than the conventional ophthalmoscope due to a novel lens system that provides a larger field of vision (25 degrees) when compared to a conventional ophthalmoscope (5 degrees), even when the patient's pupil is not dilated, while still providing high magnification 11 12 ( Figures 4 C and D ). These advantages could, in theory, facilitate the general visualization of the structures of the fundus of the eye and reduce the need of mydriatic eye drops. Around 2015, the Arclight ophthalmoscope was developed by the Arclight Project in the University of St. Andrews, Scotland. It was designed to be a lightweight and cost-effective tool, rather than an improvement over the conventional ophthalmoscope. It was meant to be used in areas with low resources, such as in rural settings and low-income countries, while also providing an adequate view of the fundus and anterior eye. In addition to serving as a simple direct ophthalmoscope, the Arclight has a blue light source for fluorescein staining examination, a red colored square for color desaturation testing; it can also be adapted into an otoscope and has solar powered battery capabilities. A smartphone adaptor for the Arclight also exists, enabling the coupling of the device to a smartphone's camera for easier photographing of the exam. 13

Some studies comparing both the Arclight and the PanOptic ophthalmoscopes to the conventional ophthalmoscope model in teaching ophthalmoscopy have been performed. Hytiris et al. (2021) conducted a trial involving 40 undergraduate medical students with no prior experience in ophthalmoscopy, their study compared the use of the Arclight ophthalmoscope with the conventional ophthalmoscope. At the end of the training, the student's skills were objectively assessed by the researchers and a questionnaire was distributed to ascertain the student's personal preferences. This study found that the students who had trained with the Arclight not only performed better at the objective testing ( *p* < 0.0001), but also subjectively preferred this model over the traditional ophthalmoscope. 14 Another study conducted by McComiskie et al. (2004) involving 10 students objectively assessed the accuracy for measuring the vertical cup-to-disk ratio of the optic disk and also the personal preference of each student after training with the PanOptic ophthalmoscope or a traditional ophthalmoscope. The student's findings were compared to a benchmarked vertical cup-to-disk ratio assessed by an ophthalmologist. The PanOptic was preferred by the students ( *p* < 0.0001), mainly because it was deemed easier to use. However, there was no difference in accuracy of the student's findings when comparing the group that used a conventional ophthalmoscope and the one that used a PanOptic. 11 Ayub et al. (2021) led a study, involving 167 students, which also compared the use of the PanOptic and conventional ophthalmoscope designs as teaching tools by using a questionnaire to measure self-confidence in fundoscopy technique for assessing optic nerve, cup-to-disc ratio, and macula. The study concluded that more undergraduate students felt confident to perform fundus examinations after initial training when using a PanOptic ophthalmoscope ( *p* < 0.01 for all assessments). However, at the end of one year after training, the level of self-confidence in performing fundoscopy was higher in the group that trained the fundus examination with a conventional ophthalmoscope. 15 No studies comparing the Arclight ophthalmoscope to the PanOptic model for undergraduate teaching have been found in the literature.

As for the application of the different ophthalmoscopes in medical practice, the Kuching diabetic eye study, conducted by Tan et al. (2010), compared the use of the PanOptic to a traditional ophthalmoscope and a slit lamp biomicroscopy in screening for diabetic retinopathy in 200 patients by the same examiner. It estimated the sensitivity and specificity of the examiner's conclusion when using either device while also assessing their ease of use. The conclusion of the study was that the PanOptic model was not superior to the conventional ophthalmoscope. 16

Considering this, current evidence suggests that among the different models of direct ophthalmoscopes, newer ophthalmoscope models can make the teaching of ophthalmoscopy for undergraduate students more enjoyable and improve the student's confidence. However, there is no ophthalmoscope design that is essentially superior, and proper training and frequent examination of the fundus are still more important than the design of the ophthalmoscope itself.

### Fundoscopy Using Smartphones

The increasing availability of portable telephones and the advances in the quality of their integrated digital cameras in the last decade have made it possible (and easy) to record sharp, detailed photographic images anywhere. This was previously impossible without the use of expensive equipment and cameras. Thus, smartphone assisted ophthalmoscopy has become a feasible alternative to traditional approaches to fundus imaging, especially in locations with limited resources. Additionally, smartphones have tools that make it possible to store and share the images obtained instantly with other physicians over the internet. This offers evident advantages over recording and sharing a purely verbal description of the findings. Smartphone fundoscopy can also be applied for the evaluation of patients who are bedbound, unable to visit a specialized outpatient clinic, or when a specialized ophthalmology clinic is unavailable. 17

There are several ways to obtain fundus images with the aid of smartphones. Different approaches imply more or less technical difficulty, while also influencing the sharpness of the image obtained and the degree of magnification of the eye's internal structures. The simplest way to photograph the structures of the eye fundus is to bring the smartphone camera close to an ophthalmoscope's eyepiece during the examination. However, the use of a conventional ophthalmoscope to obtain fundoscopy photographs implies difficulty when aligning it with the optical axis connecting the retina and the digital camera. This approach also provides the aforementioned small field of vision (5 degrees), albeit at a high magnification (15x) ( Figures 4 A and B ). Using a similar technique, the PanOptic ophthalmoscope can also be used with a smartphone. The provided silicon eyepiece of this ophthalmoscope facilitates the fixation on the patient's face, enabling a somewhat easier lens alignment, while also providing an image with a larger field of vision, easily capturing the entire optic disk and the surrounding retina ( Figures 4 C and D ).

Fundus photography using direct ophthalmoscopes can be made substantially easier with appropriate smartphone mounts that quickly align the ophthalmoscope's eye window with the camera. Considering this, several manufacturers have developed smartphone coupling systems to mitigate the technical difficulties of ophthalmoscopy using smartphones. A basic mount is available for the Arclight ophthalmoscope, which can be fixed to the back of any smartphone, enabling a fast alignment of the smartphone's camera with the ophthalmoscope lens. 13 More robust examples of such coupling systems include the iExaminer (Welch Allyn, Inc., NY, USA), which consists of a plastic mount and a separate, standalone smartphone that attaches to a PanOptic ophthalmoscope. 18 The use of the iExaminer for fundoscopic evaluation was approved in the United States of America by the FDA (Food and Drug Association) in 2013. 17 Several other systems involving smartphones exist internationally, such as the EyePhotoDoc (EyePhotoDoc, CA, USA), 19 Keeler MIO (Keeler, Windsor, UK), 20 and the Smartphone-Based Retinal Screening System from D-EYE (D-EYE, Srl., Padova, PD, Italy). 21 These, however, are not easily found in Brazil. In our territory, the Eyer (Phelcom Technologies SA, São Carlos, SP, Brazil) had its use approved by the Brazilian Health Regulatory Agency (ANVISA) in 2019. 22 23 It also consists of a standalone smartphone coupled to a system of external lenses, aligning them to the device's integrated camera. The Eyer allows for an evaluation of the anterior and posterior chambers of the eye, even through an undilated pupil. All of these systems provide more convenience for performing ophthalmoscopy and usually have their own proprietary software to save and organize the images, but those benefits imply higher costs when compared to simply using one's own smartphone with an ophthalmoscope or a condensing lens.

Smartphone assisted indirect ophthalmoscopy using condensing lenses is a technique that is becoming popular in neurology. It can be done using almost any smartphone that has a build-in camera and LED lights, and requires very little training. The smartphone must be held in one hand by the examiner, while the other should hold the lens, placing it 4 to 5 cm in front of the patient's eye. Pupil dilation with mydriatic eye drops is mandatory for this technique. The smartphone's camera, with its LED light turned on, should be positioned on the same axis connecting the patient's pupil and the condensing lens at a distance of approximately 30 cm in order to capture sharp images of the eye's fundus. In indirect ophthalmoscopy, the image obtained is inverted both in the horizontal and vertical axes. Recently, in 2021, Kohler et al. investigated this method and concluded that it is not inferior to direct ophthalmoscopy for identifying the structures of the eye fundus, even suggesting that it could be incorporated into undergraduate medical education. 24

Besides being relatively easy to perform and cost-effective, smartphone assisted indirect ophthalmoscopy using condensing lenses easily results in images that are suitable for remote expert assessment, that is to say, the practicing of telemedicine in the evaluation of the retina and optic disc. In social programs working in remote areas, where there is a lack of medical specialists or where non-portable devices such as retinographics are unavailable, obtaining fundus images from cell phones and sharing them with specialists allows for better evaluation and more complete therapeutic planning, even when a specialist cannot be present on site. 17 Online sharing of the images obtained directly from the cellphone used is also possible, allowing for the discussion of clinical cases with medical colleagues from various specialties over the globe. This facilitates the exchange of expert opinions on doubtful cases and promotes medical education.

Some studies demonstrate good diagnostic accuracy in smartphone assisted indirect fundoscopy for screening of retinopathies. 17 Unsurprisingly, when compared with conventional direct ophthalmoscopy, this method was preferred among medical students for the teaching of ophthalmology. 24 25 However, no studies validating fundoscopy performed with smartphones in the evaluation of optic neuropathies or papillaedema were identified in the literature, indicating a gap regarding this subject.

## CONCLUSION

Like any method, the capture of fundus images through smartphone cameras has disadvantages that should be noted. Among these, we can mention the greater discomfort for the patient during the exam, due to the mandatory use of mydriatic eye drops and the intensity of the LED light coming from the cellphone reaching the retina when performing indirect fundoscopy. We must also consider the variable resolution and sharpness of the images obtained, as the quality depends on the technical aspects of the smartphone used. Another important consideration is that dynamic phenomenon in the eye fundus, such as the venous pulse, while recordable with video imaging, are more difficult to observe through a smartphone camera when compared to direct ophthalmoscopy. Lastly, most existing smartphone adapters for ophthalmoscopes on the market are not universal, not allowing an easy replacement of the provided smartphone.

In conclusion, in just over 170 years of evolution, the ophthalmoscope has evolved through numerous variations, and while medical students tend to prefer newer models for the teaching of ophthalmoscopy, there is no objectively superior model for the clinical practice to date. Physicians and medical students alike find the practice of direct ophthalmoscopy difficult to master, and non-specialists tend to not perform fundoscopy in their daily practice. Moreover, the mere verbal recording of direct ophthalmoscopy findings is unsatisfactory, as it does not allow for a posterior visual comparative analysis by different professionals and makes it harder to share findings for case discussions.

With the refinement in the quality of integrated cameras in smartphones, the use of a smartphone coupled with different direct ophthalmoscopes or with a 20D lens to capture eye fundus images has emerged as a feasible modality of ophthalmoscopy. This last approach is superior to merely photographing the fundus through a direct ophthalmoscope, due to the easier alignment of the condensing lens with the patient's eye and the camera. It also provides a larger field of vision of the fundus.

Indirect ophthalmoscopy recording is also very simple to implement and learn, cost-efficient, and allows the use of the vast majority of smartphone models without the need for specific equipment or smartphone mounts. The images obtained are easily shared online between physicians and allow adequate identification of the structures of the fundus, enabling a comparative evolutionary analysis. The possibility of smartphone fundoscopy using condensing lenses (or low-cost ophthalmoscopes such as the Arclight) is especially attractive in locations with limited infrastructure, where retinographics and other specialized equipment would not be readily available. The disadvantages of indirect smartphone fundoscopy include greater discomfort to the patient due to the stronger light intensity and due to the need of mydriatic eye drops, as well as greater difficulty in assessing subtle dynamic phenomena such as venous pulse through a video recording.

Nevertheless, at present, there are few studies regarding the use of this new fundoscopy modality in the context of neurological diseases. The validation of indirect ophthalmoscopy using smartphones and condensing lenses as ophthalmoscopy techniques in neurological practice, as well as the assessment of sensitivity and specificity of the findings when compared to traditional direct ophthalmoscopy, are topics that warrant randomized clinical trials in the near future.
